# The association between the number of teeth and frailty among older nursing home residents: a cross-sectional study of the CLHLS survey

**DOI:** 10.1186/s12877-022-03688-y

**Published:** 2022-12-30

**Authors:** Xiao-Ming Zhang, Jing Jiao, Jing Cao, Xinjuan Wu

**Affiliations:** Department of Nursing, Chinese Academy of Medical Sciences Peking Union Medical College, Peking Union Medical College Hospital (Dongdan Campus), Beijing, China

**Keywords:** Frailty, Tooth loss, Toothbrushing, Frailty, Nursing home, Older adults

## Abstract

**Background:**

Given that few studies have explored the association between oral health and frailty among older nursing home residents, the purpose of this study was to assess the association between oral health (i.e., the number of teeth and oral behaviors) and frailty in this population using the Chinese Longitudinal Healthy Longevity Survey (CLHLS).

**Methods:**

This was a national cross-sectional study derived from the seventh wave of CLHLS in 2018, consisting of 365 older nursing home residents aged 65 years or older. The frailty index was constructed based on 32 variables consisting of self-rated health status, anxiety, depression, ADL and IADL. Oral health was measured through the number of natural teeth and tooth brushing behavior. Multiple logistic regression was used to identify this association between the number of teeth, oral health behaviors, and frailty.

**Results:**

The mean age of this sample was 87.6 (SD = 9.5), with 154 (42.2%) males. The prevalence of frailty and edentulism was 71.2% and 33.4%, respectively. Multiple logistic regression analysis found that the likelihood of frailty decreased with an increased number of teeth, with an OR of 0.94 (95% CI: 0.91–0.98). Compared with participants with edentulism, older adults with 1 to 20 teeth had a lower likelihood of frailty (OR = 0.39, 95% CI: 0.17–0.88); these results were also found in older adults with more than 20 teeth (OR = 0.20, 0.07–0.57). Additionally, older adults who brush their teeth regularly have a lower likelihood of frailty than those who never brush their teeth (OR = 0.37, 95% CI: 0.13–0.99).

**Conclusion:**

Older nursing home residents who maintain their natural teeth can help lower the risk of frailty, and regular toothbrushing also contributes to decreasing the risk of frailty. Our study emphasizes the importance of oral health, and cohort studies with large-scale samples to address this important issue are warranted in the future.

**Supplementary Information:**

The online version contains supplementary material available at 10.1186/s12877-022-03688-y.

## Introduction

The number of older adults is rapidly increasing worldwide, especially Chinese older adults. According to recent statistics from China, the number of older adults reached almost 253 million, accounting for 18.0% of the total Chinese population [1]. Therefore, providing high-quality care to these groups is a huge challenge for the government and authorities. In China, caring for and supporting older people is very important, and Chinese older adults mostly live in the community. Meanwhile, supported by government policy and social associations, some older Chinese adults reside in nursing homes.

Older nursing home residents often suffer from frailty, a common geriatric syndrome characterized by decreased physiological reserve and high vulnerability to insult [2]. The prevalence of frailty among older adults ranges from 10% in the community [3] to 52.3% in nursing homes [4], and frailty can lead to a high risk of adverse outcomes, for instance, falls, fractures and even mortality [5, 6]. Numerous studies have shown that identifying the risk of frailty at an early stage and implementing interventions can potentially improve frailty and sometimes even reverse it [7, 8].

Oral health is an important component of maintaining a high quality of life among older adults [9]. Oral diseases such as periodontal disease and caries are prevalent among older adults, resulting in tooth loss, which impairs daily life [10, 11]. In 2013, Andrade and colleagues explored the association between oral health and frailty among older community-dwelling adults, and the results showed that participants with 20 or more teeth had a lower likelihood of being frail than those with edentulism [12]. In recent decades, a growing body of studies have indicated that tooth loss leads to a high risk of frailty in both cross-sectional and prospective cohort studies [13–17]. In addition, studies have also found that poor oral behavior can lead to a high risk of frailty among community-dwelling older adults. However, based on recent evidence, most of these studies focused on community-dwelling older adults, with a paucity of literature investigating nursing home residents. In 2021, Saarela [18] conducted a study among older nursing home residents in Finland, and the results indicated that the difference in the number of teeth between the frail and nonfrail groups did not reach statistical significance. Therefore, more studies are needed to explore this important issue. The purpose of our study was to identify the association between the number of teeth, oral behavior, and frailty among older adults in nursing homes by using a cross-sectional study of the Chinese Longitudinal Healthy Longevity Survey. In our study, we hypothesize that nursing home older adults with fewer teeth could have a higher likelihood of being frail.

## Methods

### Data sources and setting

The data in the present study were derived from the seventh wave of the Chinese Longitudinal Health Longevity Survey (CLHLS) in 2018 [19]. The CLHLS is a national survey for Healthy Aging to explore the impact of common health-related factors on outcomes among Chinese people. This survey collected information consisting of demographic data, social and economic status, self-assessment of health-related quality, number of teeth, oral health behaviors, cognitive function, depressive symptoms, anxiety, performance in activities of daily living, chronic disease, and drugs. Detailed information about CLHLS has been published previously [20].

### Sample

The 2018 wave of CLHLS collected over 15,000 participants aged 65 years or older. Of these, 12,411 were the first to participate in an interview in 2018. In this study, we only focused on older adults who live in nursing homes. We deleted the samples with any variables that had missing values. A total of 371 older nursing home residents were included for analysis. In addition, we excluded participants who had dementia syndrome (6 nursing home residents), resulting in 365 individuals in our final analysis.

### Older nursing home residents

Nursing home residents were verified by asking the question “Who do you live with?” The participants could respond: (1) with a family member; (2) alone; (3) in a nursing home. We selected older adults who resided in a nursing home.

### Definition of the frailty index

There are two theoretical models for defining frailty—the physical frailty phenotype and the cumulative health deficit index. In this study, we used several variables to construct a frailty index. There is no consensus on how to build a frailty index, with values ranging from 30 to 70 and with a total value between 0 and 1. According to a previously published study [21], we adopted 32 indicators to calculate the frailty index, including self-rated health status, self-rated anxiety scale, the Center for Epidemiological Studies-Depression and two other scales—ADL and IADL—to assess performance in activities of daily living. Detailed information about the calculated scores for the 32 indictors is shown in Supplementary Table S1. The final frailty index is equal to the total score of 32 items divided by 32 to obtain the results for each older adult. In addition, we classified the frailty index into two categories: nonfrail (FI ≤ 0.21) and frail (FI > 0.21).

### Oral health indicators

Oral health includes the natural number of teeth, oral health behavior, and false teeth (yes versus no). Oral health behavior was defined as how often the older adult brushed their teeth every day. When participants could not hear or understand the question, the investigators asked the nursing assistant to obtain the real information. The answers consisted of “do not brush”, “occasionally”,” once a day”, “twice a day” and “three or more times a day”. We combined the answer of “do not brush” and “occasionally” into no brushing; “once a day”, “twice a day” and “three or more times a day” were categorized as regular toothbrushing.

### Covariates definition

Previous studies have reported factors that are potentially associated with frailty. We adopted variables including basic demographic data, lifestyle behaviors, examination data and the number of chronic diseases. The demographic data included age, years of education, gender, marital status, and financial support. Age was categorized as 75 years older or more and younger than 75 years; education was grouped into no education or more than 1 year of education; financial support determined whether they had sufficient finances; and marital status was classified into three categories (married, single and divorced). Regarding lifestyle behaviors, we extracted smoking, alcohol consumption, regular exercise, and regular physical labor; all of these lifestyle behaviors were categorized as yes versus no. Examination data included body mass index and calf circumference. The modified Katz index, consisting of bathing, feeding, continence, dressing, toileting, and indoor transferring, was adopted to assess ADL, and the modified Katz index was a 3-point scale (1 for without assistance, 2 for one part assistance, 3 for more than one part assistance), ranging from 6 to 18 points. The higher the ADL score is, the higher the degree of care dependency. In the CLHLS survey, participants were asked whether they had chronic diseases; 24 diseases were included. Information on visual impairment and hearing loss was also collected. Cognitive function was assessed by the Chinese Mini-Mental State Examination (MMSE).

### Data analysis

We used descriptive analysis to present the data with frequencies or percentages and means (standard deviations) when the data were continuous and categorical variables, respectively. Analysis of variance and the chi-square test were used to compare the differences in terms of variables among the three groups (0 teeth, 1 to 20 teeth, and > 20 teeth). Univariate logistic regression was adopted to identify the factors associated with frailty status using the crude OR. Potentially confounding factors were based on clinical factors related to frailty; the number of teeth was the independent variable, and the frailty status was the dependent variable. First, the association between the number of teeth and frailty was identified by a generalized additive model (GAM) analysis to confirm whether there was a nonlinear relationship between the number of teeth and frailty. Multiple logistic regression was employed to identify the independent association between the number of teeth or teeth group and frailty after adjusting for potential confounding factors. Two models were conducted for this cross-sectional association between the number of teeth and frailty. Model 1 was listed without any adjustment, and model 2 was adjusted for sex, age group, years of education, drinking, false teeth, visual impairment, hearing loss, cognitive impairment, sufficient financial support, and exercise. Finally, we also performed a sensitivity analysis after deleting the lowest 10% frailty status scores to confirm whether the association between the number of teeth and frailty still existed according to a previous study [22].

## Results

### Baseline characteristics of the total sample

There were 365 older nursing home residents included in this study, of which the mean age was 87.6 (SD = 9.5), with 154 (42.2%) males. The majority of the total sample was widowed (75.4%), and over half of the participants were illiterate (55.7%). The mean ADL score in this sample was 7.9 (SD = 3.0). The prevalence of edentulism was 33.4%, and 56 (15.3%) had more than 20 teeth. The prevalence of frailty was 71.2%, and the mean cognitive function was 23.2 (SD = 6.6). In terms of lifestyle behaviors, 9.5% smoked, and 9.4% drank. Only 36.4% of participants engaged in regular exercise. The prevalence of visual impairment and hearing loss was 20.9% and 42.7%, respectively. Regarding the main diagnosis, the prevalence rates of hypertension, diabetes and heart disease were 58.3%, 16.8% and 34.9%, respectively (Table 1).

**Table 1:** Baseline of characteristics among total sample and different teeth groups

Variables	Total (*N* = 365)	Teeth = 0 (*N* = 122)	Teeth =1–20 (*N* = 187)	Teeth > 20 (*N* = 56)	*P*-value
Age (mean, SD)	87.6 ± 9.5	92.7 ± 7.7	86.4 ± 9.0	80.3 ± 8.6	< 0.001
number of teeth (mean, SD)	8.9 ± 9.8	0.0 ± 0.0	9.4 ± 5.9	27.0 ± 2.6	< 0.001
MMSE (mean, SD)	23.2 ± 6.6	20.9 ± 7.6	23.9 ± 5.9	25.7 ± 5.0	< 0.001
Calf circumference (mean, SD)	31.7 ± 6.1	31.8 ± 6.3	31.6 ± 6.1	32.0 ± 5.8	0.688
ADL scores (mean, SD)	7.9 ± 3.0	8.5 ± 3.3	7.7 ± 2.9	7.4 ± 2.7	0.032
Age group (n %)					< 0.001
< 75	34 (9.3%)	2 (1.6%)	19 (10.2%)	13 (23.2%)	
> = 75	331 (90.7%)	120 (98.4%)	168 (89.8%)	43 (76.8%)	
Sex (n %)					0.529
Male	154 (42.2%)	56 (45.9%)	77 (41.2%)	21 (37.5%)	
Female	211 (57.8%)	66 (54.1%)	110 (58.8%)	35 (62.5%)	
Marital statue (n %)					0.013
Married	62 (17.4%)	11 (9.5%)	38 (20.5%)	13 (23.2%)	
Divorced	8 (2.2%)	2 (1.7%)	2 (1.1%)	4 (7.1%)	
Widowed	269 (75.4%)	95 (81.9%)	137 (74.1%)	37 (66.1%)	
Never married	18 (5.0%)	8 (6.9%)	8 (4.3%)	2 (3.6%)	
Education group (n %)					< 0.001
0 year	190 (55.7%)	76 (65.0%)	98 (56.0%)	16 (32.6%)	
> = 1 year	151 (44.3%)	41 (35.0%)	77 (44.0%)	33 (67.4%)	
Hypertension (n %)					0.079
No	144 (41.7%)	56 (49.1%)	71 (40.1%)	17 (31.5%)	
Yes	201 (58.3%)	58 (50.9%)	106 (59.9%)	37 (68.5%)	
Diabetes (n %)					0.604
No	258 (83.2%)	81 (84.4%)	137 (84.1%)	40 (78.4%)	
Yes	52 (16.8%)	15 (15.6%)	26 (15.9%)	11 (21.6%)	
Heart disease (n %)					0.326
No	207 (65.1%)	67 (65.0%)	110 (67.9%)	30 (56.6%)	
Yes	111 (34.9%)	36 (35.%)	52 (32.1%)	23 (43.4%)	
Stroke or Cardiovascular disease (n %)					0.324
No	249 (79.3%)	83 (83.0%)	129 (79.1%)	37 (72.5%)	
Yes	65 (20.7%)	17 (17.0%)	34 (20.9%)	14 (27.5%)	
Number of comorbidities (median, IQR)	2.00 (1.00–4.00)	2.00 (1.00–5.00)	(1.00–4.00)	3.00 (1.00–5.00)	0.340
Sufficient finance support (n %)					0.576
Yes	325 (89.3%)	106 (87.6%)	167 (89.3%)	52 (92.9%)	
No	39 (10.7%)	15 (12.4%)	20 (10.7%)	4 (7.1%)	
Smoking (n %)					0.590
Yes	34 (9.5%)	12 (9.9%)	15 (8.2%)	7 (12.7%)	
No	325 (90.5%)	109 (90.1%)	168 (91.8%)	48 (87.3%)	
Drinking (n %)					0.395
Yes	34 (9.4%)	15 (12.3%)	15 (8.1%)	4 (7.3%)	
No	328 (90.6%)	107 (87.7%)	170 (91.9%)	51 (92.7%)	
False teeth (n %)					< 0.001
Yes	165 (45.3%)	82 (67.8%)	66 (35.3%)	17 (30.4%)	
No	199 (54.7%)	39 (32.2%)	121 (64.7%)	39 (69.6%)	
Visual impairment (n %)					< 0.001
NO	287 (79.1%)	80 (66.1%)	158 (84.5%)	49 (89.1%)	
Yes	76 (20.9%)	41 (33.9%)	29 (15.5%)	6 (10.9%)	
Difficulty with hearing (n %)					< 0.001
Yes	156 (42.7%)	72 (59.0%)	68 (36.4%)	16 (28.6%)	
No	209 (57.3%)	50 (41.0%)	119 (63.6%)	40 (71.4%)	
Exercise (n %)					0.108
Yes	132 (36.4%)	36 (29.8%)	71 (38.0%)	25 (45.5%)	
No	231 (63.6%)	85 (70.2%)	116 (62.0%)	30 (54.5%)	
Physical labor regularly (n %)					0.030
Yes	179 (50.3%)	70 (58.3%)	89 (48.9%)	20 (37.0%)	
No	177 (49.7%)	50 (41.7%)	93 (51.1%)	34 (63.0%)	
Falls in the past 1 year (n %)					0.872
Yes	73 (20.2%)	24 (19.7%)	39 (21.2%)	10 (18.2%)	
No	288 (79.8%)	98 (80.3%)	145 (78.8%)	45 (81.8%)	
Brush your teeth (n %)					0.012
No	79 (22.1%)	35 (30.2%)	38 (20.4%)	6 (10.7%)	
Yes	279 (77.9%)	81 (69.8%)	148 (79.6%)	50 (89.3%)	
Nutrient supplements (n %)					0.365
Yes	64 (18.0%)	19 (16.8%)	38 (21.0%)	7 (13.0%)	
No	290 (81.7%)	100 (83.2%)	143 (79.0%)	47 (87.0%)	
Frailty (n %)					< 0.001
No	105 (28.8%)	19 (15.6%)	59 (31.5%)	27 (48.2%)	
Yes	260 (71.2%)	103 (84.4%)	128 (68.5%)	29 (51.8%)	

### Baseline characteristics of the different teeth groups (number = 0; 0–20; ≥21)

Compared to older people with natural teeth, edentulous individuals were older (mean age: 92.7 versus 86.4 versus 80.3; *p* < 0.001) and more likely to be illiterate (65.0%), have more false teeth (67.8%), suffer from visual impairment (33.9%) and hearing loss (59.0%), have lower cognitive function (20.9 ± 7.6), have a high degree of care dependency, and be more likely to be frail (84.4%). In contrast, there were no differences in terms of sex, sufficient financial support, smoking, drinking, calf circumference, BMI, falling history, number of comorbidities, exercise, or nutrient supplementation between these teeth categories (both *p* > 0.05) (Table 1).

### Univariate analysis between independent factors and frailty

Table 2 summarizes the unadjusted analyses of the association between variables and frailty. The results found that frail nursing residents were older, female, had fewer teeth, had lower cognitive function and were unlikely to exercise. There were significant differences in terms of variables such as visual dysfunction, hearing loss, brushing teeth, calf circumference, and years of education (both *p* < 0.05).

**Table 2:** Univariate analysis between independent factors and frailty

	Statistics	Frailty OR(95%CI)	*P*-value
Age (mean, SD)	87.6 ± 9.5	1.09 (1.06, 1.12)	< 0.001
BMI (mean, SD)	22.8 ± 3.7	1.00 (0.94, 1.07)	0.953
Number of comorbidities (median, IQR)	2.00 (1.00–4.00)	1.02 (0.98, 1.06)	0.308
ADL scores (mean, SD)	7.9 ± 3.0	6.33 (2.82, 14.22)	< 0.001
Calf circumference (mean, SD)	31.7 ± 6.1	0.94 (0.91, 0.98)	0.001
Age group (n%)			
< 75	34 (9.3%)	Reference	
> = 75	331 (90.7%)	1.85 (0.89, 3.81)	0.097
Education group (n%)			
0 year	190 (55.7%)	Reference	
> = 1 year	151 (44.3%)	0.54 (0.34, 0.87)	0.011
Stroke or CVD (n%)			
No	249 (79.3%)	Reference	
Yes	65 (20.7%)	1.92 (0.95, 3.88)	0.069
Heart disease (n%)			
No	207 (65.1%)	Reference	
Yes	111 (34.9%)	1.10 (0.65, 1.85)	0.718
Diabetes (n%)			
No	258 (83.2%)	Reference	
Yes	52 (16.8%)	0.68 (0.36, 1.28)	0.226
Hypertension (n%)			
No	144 (41.7%)	Reference	
Yes	201 (58.3%)	0.65 (0.40, 1.05)	0.078
Marital status (n%)			
married	62 (17.4%)	Reference	
divorced	8 (2.2%)	0.77 (0.18, 3.37)	0.730
widowed	269 (75.4%)	2.42 (1.36, 4.30)	0.002
never married	18 (5.0%)	0.77 (0.27, 2.21)	0.628
Sufficient financial support (n%)			
Yes	325 (89.3%)	Reference	
NO	39 (10.7%)	1.94 (0.83, 4.56)	0.125
Smoking (n%)			
Yes	34 (9.5%)	Reference	
NO	325 (90.5%)	5.52 (2.62, 11.66)	< 0.001
Drinking (n%)			
Yes	34 (9.4%)	Reference	
NO	328 (90.6%)	4.15 (2.01, 8.58)	0.001
Sex (n%)			
Male	154 (42.2%)	Reference	
Female	211 (57.8%)	1.79 (1.13, 2.83)	0.012
number of teeth (mean, SD)	8.9 ± 9.8	0.95 (0.93, 0.97)	< 0.05
False teeth (n%)			
Yes	165 (45.3%)	Reference	
NO	199 (54.7%)	1.41 (0.90, 2.23)	0.137
Visual impairment (n%)			
No	287 (79.1%)	Reference	
Yes	76 (20.9%)	2.52 (1.29, 4.89)	0.006
Difficulty with hearing (n%)			
Yes	156 (42.8%)	Reference	
NO	209 (57.2%)	0.29 (0.17, 0.48)	< 0.001
MMSE	23.23 ± 6.67	0.78 (0.72, 0.84)	< 0.001
Exercise (n%)			
Yes	132 (36.4%)	Reference	
NO	231 (63.6%)	4.49 (2.77, 7.28)	< 0.001
Physical labor regularly (n%)			
Yes	179 (50.3%)	Reference	
No	177 (49.7%)	0.93 (0.59, 1.48)	0.763
Falls in the past 1 year (n%)			
No	73 (20.2%)	Reference	
Yes	288 (79.8%)	0.70 (0.39, 1.28)	0.245
Brushing teeth (n%)			
No	79 (22.1%)	Reference	
Yes	279 (77.9%)	0.18 (0.08, 0.41)	< 0.001
Nutrient supplements (n%)			
Yes	64 (18.0%)	Reference	
No	290 (81.7%)	1.77 (1.01, 3.12)	0.047
Number of teeth categories (n%)			
<=0	122 (33.4%)	Reference	
> 0, <=20	187 (51.2%)	0.40 (0.22, 0.71)	0.002
> 20	56 (15.4%)	0.20 (0.10, 0.41)	< 0.001

### Nonlinear relationship analyses

**Fig. 1:**
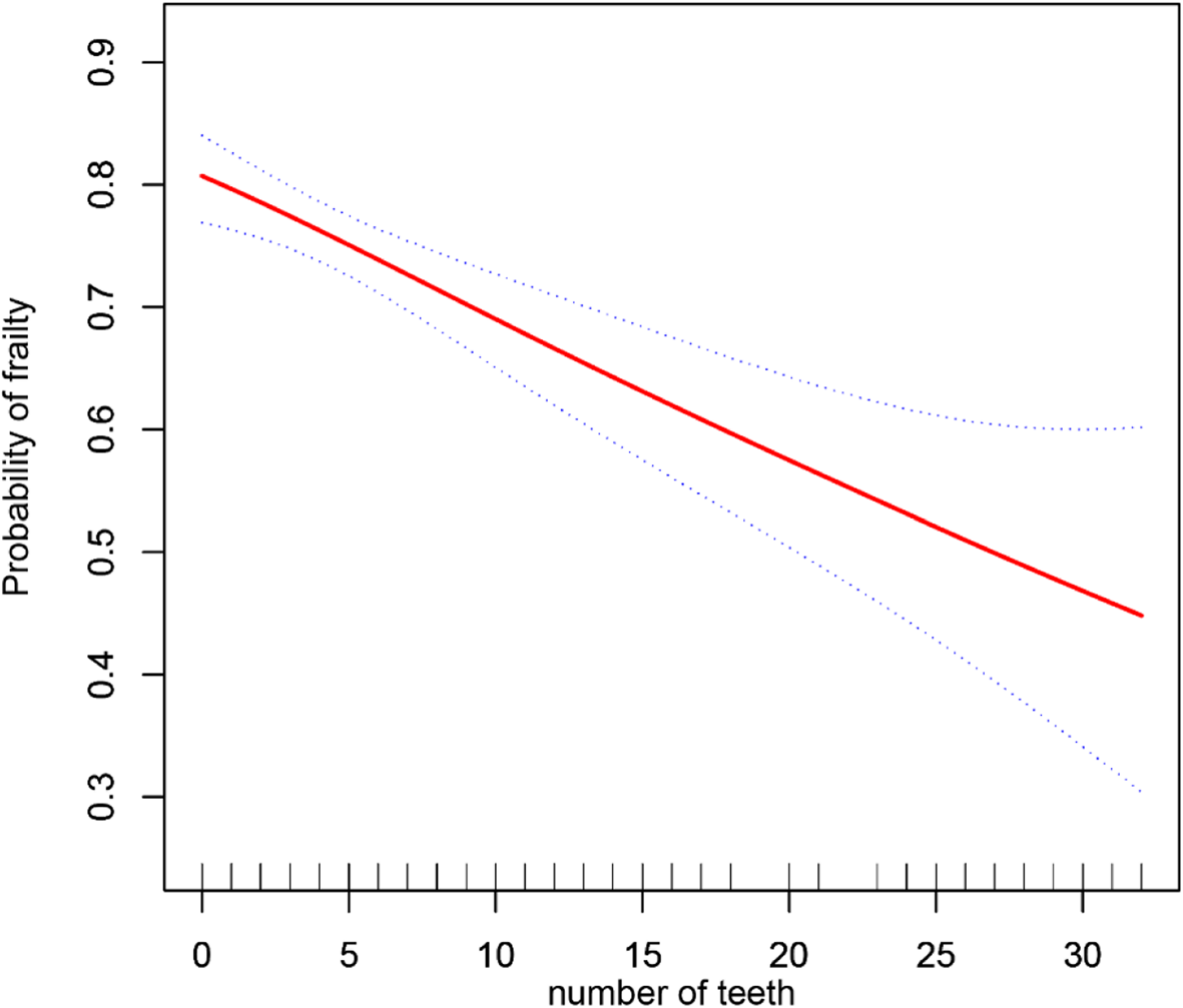
A generalized additive model shows a linear association between the number of teeth and frailty

The results of the GAM analysis found that there was a linear relationship between the number of teeth and frailty. The probability of frailty decreased when the number of teeth among older nursing home residents increased, as shown in Fig. 1.

### Multiple logistic regression between tooth number, Toothbrushing, and frailty

The results of the multiple logistic regression analysis for different adjustments are shown in Table 3. There was a significant association between tooth number and the likelihood of frailty in the unadjusted model (OR = 0.95, 95% CI: 0.93, 0.97). After fully adjusting for potential factors, including sex, age group, years of education, drinking, false teeth, visual impairment, hearing loss, cognitive impairment, sufficient financial support and exercise, the association still existed, with an OR of 0.94 (95% CI: 0.91–0.98) (Table 3a). When categorizing the number of teeth into three classifications, compared to older adults with edentulism, individuals with 1 to 20 teeth had a lower likelihood of frailty (OR = 0.39, 95% CI: 0.17, 0.88), and individuals with more than 20 teeth also had a lower likelihood of frailty (OR = 0.20, 95% CI: 0.07, 0.57) in the fully adjusted model (Table 3b). Furthermore, older adults who performed regular toothbrushing had a lower risk of frailty than those who did not brush their teeth in the unadjusted model (OR = 0.20, 95% CI: 0.09–0.47) and adjusted model (OR = 0.37, 95% CI: 0.13–0.99).

**Table 3a:** The association between the number of teeth and frailty according to multiple logistic regression analysis in different models

	Crude model OR, 95%CI	*P*-value	Adjusted model1 OR,95%CI	*P*-value
Number of teeth	0.95(0.93–0.97)	< 0.05	0.94(0.91–0.98)	< 0.05
Brush your teeth				
No	Reference		Reference	
Yes	0.20(0.09–0.47)	< 0.05	0.37(0.13–0.99)	0.04
Age group				
< 75			Reference	
> = 75			0.93(0.36–2.39)	0.88
Sex				
Male			Reference	
Female			1.83(0.96–3.47)	0.06
Hearing difficult				
Yes			Reference	
No			0.38(0.19–0.73)	< 0.05
Exercise				
Yes			Reference	
NO			2.59(1.42–4.71)	< 0.05
Sufficient finance support				
Yes			Reference	
No			1.95(0.64–5.93)	0.23
Visual impairment				
NO			Reference	
Yes			2.34(0.94–5.85)	0.06
Education group				
0 year			Reference	
> = 1 year			1.56(0.82–2.96)	0.16
Cognitive impairment				
No			Reference	
Yes			3.97(1.78–8.85)	< 0.05
False teeth				
Yes			Reference	
No			1.30(0.68–2.49)	0.41
Drinking				
Yes			Reference	
No			2.29(0.83–6.34)	0.10

**Table 3b:** The association between teeth categories and frailty according to multiple logistic regression analysis in different models

	Crude model OR,95%CI	*P*-value	Adjusted model1 OR,95%CI	*P*-value
Teeth categories				
=0	Reference		Reference	
> 0, <=20	0.45(0.25–0.82)	< 0.05	0.39(0.17–0.88)	0.02
> 20	0.25(0.12–0.52)	< 0.05	0.20(0.07–0.57)	< 0.05
Brush your teeth				
No	Reference		Reference	
Yes	0.20(0.09–0.46)	< 0.05	0.37(0.14–0.99)	0.04
Age group				
< 75			Reference	
> = 75			0.95(0.37–2.44)	0.93
Sex				
Male			Reference	
Female			1.83(0.96–3.48)	0.06
Hearing difficult				
Yes			Reference	
No			0.38(0.19–0.74)	< 0.05
Exercise				
Yes			Reference	
NO			2.63(1.44–4.78)	< 0.05
Sufficient finance support				
Yes			Reference	
No			1.95(0.66–5.78)	0.23
Visual impairment				
NO			Reference	
Yes			2.18(0.87–5.43)	0.09
Education group				
0 year			Reference	
> = 1 year			1.57(0.82–2.99)	0.16
Cognitive impairment				
No			Reference	
Yes			4.03(1.81–8.97)	< 0.05
False teeth				
Yes			Reference	
No			1.36(0.70–2.63)	0.35
Drinking				
Yes			Reference	
No			2.19(0.78–6.18)	0.13

### Sensitivity analysis

A cross-sectional study usually has a high risk of reverse causality. We conducted a sensitivity analysis by removing the lowest 10% frailty scores. A total of 25 individuals with the lowest frailty scores were excluded, and we conducted multiple logistic regressions with the remaining data. The results showed that after adjusting for the same confounding factors mentioned above, the number of teeth was still associated with a lower likelihood of frailty among older nursing home residents (OR = 0.93, 95% CI: 0.90–0.97. In addition, participants who brushed their teeth regularly had a lower likelihood of frailty than those who did not brush their teeth (OR = 0.21, 95% CI: 0.05–0.77) (Supplementary Table S2).

## Discussion

In the present study, we found that the number of teeth was associated with a lower risk of frailty. Older nursing home adults who brush their teeth have a lower risk of frailty than individuals who do not brush their teeth as part of their daily life. Our study emphasizes the importance of maintaining teeth and brushing teeth, which could reduce the risk of frailty among residents.

In our study, the prevalence of edentulism was 33.3% among older nursing home residents, which was higher than that in community-dwelling older adults reported in two previous studies (19.4% [23] and 23.5% [24]). The main reason for this discrepancy was that the mean age in our study was higher than that in the abovementioned previous study (87.6 years versus 77.2). When adults become aging, they often suffer from some oral disease, which leads to tooth loss [25]. A previous study conducted in the United States reported that the edentulism prevalence among participants aged 65–74 and ≥ 75 was 15% and 22%, respectively [26]. Nursing home residents with edentulism can easily develop frailty because of deficient nutrition intake [27].

Our study found that there was a significant association between tooth loss and a greater likelihood of frailty among older nursing home residents, which was in line with previous studies. In a study covering 903 community-dwelling older adults, the number of teeth was associated with frailty, with an OR = 0.98 (95% CI: 0.96–0.99) [17]. In addition, a cohort study with 3 years of follow-up among home-dwelling individuals aged 70 or older indicated that one additional tooth could lower the risk of frailty by 5.0% [14]. Although there are a growing body of studies consisting of cross-sectional or cohort studies investigating the association between tooth loss and frailty, to date, only one published study has investigated this association in a nursing home setting. In 2021, Saarela et al. conducted research to explore the association between oral health and frailty among older adults living in long-term care [18]. The results showed that the mean number of teeth between frail residents and nonfrail residents was not significantly different, with a figure of 9.7 (9.3) versus 11.0 (9.3), which was inconsistent with our study. There are a few reasons that could explain this difference between our current study and that of Saarela and colleagues. First, our study included a nationwide sample of nursing home residents, whereas theirs studied a single center; this might reduce selection bias. Second, the sample size in Saarela’s study was smaller than in ours, which might fail to reach enough statistical power to detect the difference. However, a large-scale study is needed to confirm the association between the number of teeth and frailty among older nursing home residents in the future.

Our study also found that the oral health indicator, brushing teeth regularly, could reduce the likelihood of frailty in nursing homes. One of the reasons for teeth loss among older adults was poor health behaviors, such as failing to brush regularly. A previous study conducted among older nursing home adults aged 75 years indicated that the proportion of oral cleaning habits was lower among frail dentate people than among those without frailty, with a figure of 52.7% versus 75.4%, which was similar to our present study [28]. In addition, in another study exploring the association between the oral environment and frailty, the results found that frailty tends toward a higher proportion of poor hygiene [29]. Regular toothbrushing can help maintain good hygiene and clean away harmful bacteria, which helps maintain good oral health, reducing the likelihood of frailty among older adults. However, we need to be cautious because frailty itself might also affect older adults’ ability to hold a toothbrush. Those frail older adults were more likely to have impaired health, declined performance in activities of daily living, and chronic disease, which contributes to poor oral behaviors and lower dental service use. A previous qualitative study indicated that institutionalized older adults suffered from poor oral health because they were disoriented or lacked support from family, friends, and society [30].

The mechanism of tooth loss in developing frailty has been well described before and involves three potential pathways: nutritional, psychological, and inflammatory factors [31, 32]. Older adults with tooth loss, especially edentulism without false teeth, have to ensure they have a varied diet and are more likely to have to eat soft food because of their decreased chewing ability [33]. When older adults endure this condition for a period of time, they may suffer from frailty through malnutrition. In addition, older adults with tooth loss might have less confidence when communicating with others, which leads to lower levels of physical activity [34] and a higher likelihood of depression [35], ultimately contributing to frailty. More studies need to confirm these three mechanistic pathways among older nursing home residents.

Our study has many clinical implications. Due to the results supporting the maintenance of natural teeth and consistent oral health care behavior and toothbrushing, strategies and interventions to help older nursing home residents maintain good oral health are warranted. For instance, interventions such as improving the knowledge and awareness of oral health benefit older adults to protect their natural teeth. In fact, in a previous review study that summarized the current evidence on interventions for improving oral health, the results supported that educational interventions, professional oral healthcare, and restorative treatment can improve oral health among older adults [36]. However, from the preventive perspective, nursing home staff and clinicians need to adopt strategies to prevent older adults from losing their teeth. In addition, for frailty, oral health interventions combined with a traditional effective program such as exercise and nutrition need to be explored in the future.

Our study has some strengths and limitations. First, this was the first study to explore the association between oral health (number of teeth and oral behaviors) and frailty among older nursing home residents from China based on a literature review. Second, our study employed comprehensive statistical analysis, such as full adjustment, generalized additive model analysis, and sensitivity analysis, making our results reliable and credible. Third, our study has significant clinical implications for maintaining oral health among this population—older nursing home residents. However, there were some limitations that need to be taken into account. First, due to the nature of cross-sectional studies, cause-and-effect associations cannot be determined; however, we performed a sensitivity analysis by deleting the worst 10% frailty scores from the total sample. The results showed similar findings, which could help reduce the risk of reverse causality. Second, in our study, we did not construct frailty based on Physical Frailty Phenotype according to the CLHLS; thus, we cannot compare our results with previous studies that used Physical Frailty Phenotype as an assessment tool. Third, some important factors that are closely related to frailty, such as malnutrition, were not included in the CLHLS, which might overestimate the results. Fourth, a previous study confirmed that oral hygiene affected frailty among older people. However, the original Public Data did not provide this important variable, which should be explored in future studies.

## Conclusions

Our study found that the number of teeth and regular toothbrushing decreased the risk of frailty among older nursing home residents, emphasizing the importance of oral health. Our findings suggest that clinicians or staff from nursing homes need to take measures to maintain natural teeth and adopt a strategy to encourage older nursing home residents to maintain oral hygiene. Future studies consisting of cohort studies or randomized clinical trials to identify the association between oral health and frailty in older adults residing in nursing homes are warranted.

## Supplementary Information

**Additional file 1: Supplementary Table 1. **Variables in The 32-Item Frailty Index and Their Respective Scorings.

**Additional file 2:** **Supplemental Table2a.** Multiple logistic regression between oral number of teeth, and frailty after removing the worst frailty status in different models, Supplemental Table2b: Multiple logistic regression between oral teeth categories, and frailty after removing the worst frailty status in different models.

## Data Availability

All the data used in our study were obtained from a public database named Peking University Open Research Data (10.18170/DVN/WBO7LK).

## Abbreviations

CLHLS: Chinese Longitudinal Healthy Longevity Survey
ADL: activities of daily living
IADL: instrumental activities of daily living
MMSE: Mini-Mental State Examination
GAM: generalized additive model
OR: odds ratio; 95% confidence interval
