# Supplementary material for: The association between the number of teeth and frailty among older nursing home residents: a cross-sectional study of the CLHLS survey
Source: BMC Geriatr. 2022 Dec 30;22:1007. doi: 10.1186/s12877-022-03688-y (PMC9805096; doi:10.1186/s12877-022-03688-y)
Supplement: Supplementary file 1 — Additional file 1: Supplementary Table 1. Variables in The 32-Item Frailty Index and Their Respective Scorings. [file 12877_2022_3688_MOESM1_ESM.docx]

Supplemental Table2a: Multiple logistic regression between oral number of teeth, and frailty after removing the worst frailty status in different models

|  | Crude model |  | Adjusted model1 |  |
| --- | --- | --- | --- | --- |
|  | OR, 95%CI | P-value | OR,95%CI | P-value |
| Number of teeth | 0.95(0.93-0.98) | <0.05 | 0.93(0.90-0.97) | <0.05 |
| Brush your teeth |  |  |  |  |
| No | Reference |  | Reference |  |
| Yes | 0.14(0.05-0.41) | <0.05 | 0.21(0.05-0.77) | 0.02 |
| Age group |  |  |  |  |
| <75 |  |  | Reference |  |
| >=75 |  |  | 1.05(0.35-3.14) | 0.92 |
| Sex |  |  |  |  |
| Male |  |  | Reference |  |
| Female |  |  | 2.03(0.97-4.24) | 0.06 |
| Hearing difficult |  |  |  |  |
| Yes |  |  | Reference |  |
| No |  |  | 0.53(0.25-1.12) | 0.09 |
| Exercise |  |  |  |  |
| Yes |  |  | Reference |  |
| NO |  |  | 2.66(1.35-5.25) | <0.05 |
| Sufficient finance support |  |  |  |  |
| Yes |  |  | Reference |  |
| No |  |  | 5.96(1.04-33.92) | 0.04 |
| Visual impairment |  |  |  |  |
| NO |  |  | Reference |  |
| Yes |  |  | 2.58(0.90-7.43) | 0.07 |
| Education group |  |  |  |  |
| 0 year |  |  | Reference |  |
| >=1 year |  |  | 1.48(0.71-3.09) | 0.28 |
| Cognitive impairment |  |  |  |  |
| No |  |  | Reference |  |
| Yes |  |  | 5.75(1.98-16.65) | <0.05 |
| False teeth |  |  |  |  |
| Yes |  |  | Reference |  |
| No |  |  | 1.28(0.61-2.67) | 0.51 |
| Drinking |  |  |  |  |
| Yes |  |  | Reference |  |
| No |  |  | 2.33(0.74-7.30) | 0.14 |

Supplemental Table2b: Multiple logistic regression between oral teeth categories, and frailty after removing the worst frailty status in different models

|  | Crude model |  | Adjusted model1 |  |
| --- | --- | --- | --- | --- |
|  | OR,95%CI | P-value | OR,95%CI | P-value |
| Teeth categories |  |  |  |  |
| =0 | Reference |  | Reference |  |
| >0, <=20 | 0.43(0.22-0.85) | 0.01 | 0.40(0.16-1.00) | 0.05 |
| >20 | 0.22(0.10-0.52) | <0.05 | 0.17(0.05-0.57) | <0.05 |
| Brush your teeth |  |  |  |  |
| No | Reference |  | Reference |  |
| Yes | 0.14(0.05-0.40) | <0.05 | 0.21(0.06-0.79) | 0.02 |
| Age group |  |  |  |  |
| <75 |  |  | Reference |  |
| >=75 |  |  | 1.11(0.38-3.28) | 0.83 |
| Sex |  |  |  |  |
| Male |  |  | Reference |  |
| Female |  |  | 1.93(0.93-4.00) | 0.07 |
| Hearing difficult |  |  |  |  |
| Yes |  |  | Reference |  |
| No |  |  | 0.51(0.24-1.09) | 0.08 |
| Exercise |  |  |  |  |
| Yes |  |  | Reference |  |
| NO |  |  | 2.64(1.34-5.20) | <0.05 |
| Sufficient finance support |  |  |  |  |
| Yes |  |  | Reference |  |
| No |  |  | 5.59(1.03-30.2) | 0.04 |
| Visual impairment |  |  |  |  |
| NO |  |  | Reference |  |
| Yes |  |  | 2.26(0.79-6.46) | 0.12 |
| Education group |  |  |  |  |
| 0 year |  |  | Reference |  |
| >=1 year |  |  | 1.45(0.69-3.01) | 0.31 |
| Cognitive impairment |  |  |  |  |
| No |  |  | Reference |  |
| Yes |  |  | 5.48(1.90-15.73) | <0.05 |
| False teeth |  |  |  |  |
| Yes |  |  | Reference |  |
| No |  |  | 1.22(0.58-2.56) | 0.58 |
| Drinking |  |  |  |  |
| Yes |  |  | Reference |  |
| No |  |  | 2.12(0.67-6.65) | 0.19 |
